# Validation and Cultural Adaptation of an Arabic Version of Pediatric Eating Assessment Tool (Pedi-EAT-10_Arabic_)

**DOI:** 10.1007/s00455-021-10404-2

**Published:** 2022-01-11

**Authors:** Sally M. Adel, Alaa H. Gaafar, Nader Fasseeh, Rania M. Abdou, Nesrine Hazem Hamouda

**Affiliations:** 1 Phoniatrics Unit, Oto-Rhino-Laryngology Department, Faculty of Medicine, Alexandria University, Champollion Street, Alexandria Main University Hospital, El Sultan Hussein, Egypt; 2 Otorhinolaryngology Department, Faculty of Medicine, Alexandria University, Alexandria Main University Hospital, Champollion Street, El Sultan Hussein, Egypt; 3 Respiratory and Allergy Unit, Pediatrics Department, Faculty of Medicine, Alexandria University, Champollion Street, Alexandria Main University Hospital, El Sultan Hussein, Egypt

**Keywords:** Pedi-EAT 10, Arabic version, Pediatric dysphagia, Aspiration, Questionnaire, Validation, Pharyngeal residue

## Abstract

Pediatric eating assessment tool (Pedi-EAT-10_Arabic_) is a validated and reliable caregiver administered outcome instrument designed for detection of children at high risk of penetration/aspiration. The objective of this study is to translate and validate the Arabic version of Pedi-EAT-10 and to correlate its results with pharyngeal residue and aspiration on fiber optic endoscopic examination of swallowing (FEES). A cross-sectional study including 202 children selected randomly from those attending the swallowing clinic in phoniatrics unit, Otorhinolaryngology department (ORL) at main university hospital between February 2019 and October 2020 complaining of dysphagia. For test–retest reliability, one hundred caregivers refilled the Pedi-EAT-10_Arabic_ after a 2-week period following their first visit. Validity was established by comparing the scores of dysphagia patients to healthy controls. Internal consistency of Pedi-EAT-10_Arabic_ was high (Cronbach's alpha 0.986). Intra class correlation showed excellent test–retest reliability (*r* = 0.968). The median Pedi-EAT 10_Arabic_ score was significantly higher in dysphagia group compared to healthy controls. (Median 27 IQR 21–34 for cases compared to median zero IQR 0–2 points for healthy controls, *P* less than 0.001). A strong correlation was found between Pedi-EAT 10_Arabic_ scores and PAS scores with Spearman's correlation coefficient *r* = 0.803 and *P* < 0.001. The ROC for evaluating the discriminatory capacity of Pedi-EAT 10 for aspiration showed an AUC of 0.92 (95% CI of 0.89 to 0.96)_._ Conclusion: Pedi-EAT 10_Arabic_ was found to be a valid and reliable screening tool for further instrumental assessment of risk of dysphagia in pediatric population.

## Introduction

Oropharyngeal dysphagia has been recently addressed more in the pediatric population [1]. According to research, around 1% of children in the general community suffer from swallowing problems [2]. But this incidence is substantially higher in special populations of the pediatric age group (e.g. children with cerebral palsy, traumatic brain injury, and airway malformations [3, 4].

There is a high incidence of parental reporting of abnormal swallowing and feeding function up to 50% of the parents of general pediatric population and the negative impacts on children [3, 5–7]. These negative impacts include dehydration, malnutrition which consequently affects both development and psychological well-being. Pulmonary complications, namely pneumonia, pulmonary abscess, and death are also common consequences of oropharyngeal dysphagia [4, 8, 9].

Despite dysphagia was reported in healthy children, it is more common in pediatric patients with a history of prematurity, neuromuscular disorders, cardiopulmonary disorders, upper aero-digestive tract congenital anomalies, and gastrointestinal tract disorders [10].

Even though early recognition of pediatric dysphagia is important to ensure the appropriate precautions and interventions. This disorder is frequently missed in clinical practice [9]. Pediatric swallowing disorders are assessed by both non-instrumental and instrumental techniques [5]. The two most commonly used instrumental evaluations of swallowing for the pediatric population are the video fluoroscopic swallowing study (VFSS) [11, 12] and FEES [13, 14]. It is not feasible to perform these procedures on all pediatric patients at risk for dysphagia because of time, cost, and need for specialized equipment [15].

The non-instrumental assessment has evolved into an important aspect of the clinical evaluation of dysphagia to determine the need for instrumental detailed evaluation of swallowing [16, 17].

Though over referral to instrumental assessment is of concern for an adult patient, it is more concerning for the pediatric patient and further investigation is required to improve the sensitivity and specificity of a swallow screen. Extra consideration needs to be made in the frequency of radiation exposure, the risks of exposure in an infant or young child, and the ability of a child to complete the instrumental assessment. Some children may refuse to complete the swallow study due to the presence of barium in the food. Others will not tolerate placement of a scope [18]. In an effort to preserve personal protective equipment (PPE) and limit exposure of patients and clinicians to SARS-CoV-2 [19], following the guidance from the Centers for Medicare & Medicaid Services (CMS), ASHA recommended delaying non-essential endoscopies in those with unknown transmission risk [20].

The validity and reliability of the Pedi-EAT 10 have been proven in patients with a wide range of dysphagia etiologies. It has high internal consistency and test–retest reliability. This test can be used to determine the severity of dysphagia as well as track treatment progress and therapeutic effects. It's a five-point ordinal scale ranging from 0 (no problem) up to 4 (very difficult or severe problem) [15].

The use of Pedi-EAT-10 as a screening tool to determine whether a child's swallowing function requires additional instrumental evaluation has been proved. It also has the advantage of capturing dysphagia symptoms in everyday situations rather than a single point of time assessment in the clinic which can miss a lot of important information about the child’s typical swallowing function [15].

The purpose of the current study is to develop an Arabic version of Pedi-EAT 10 questionnaire, and to test its validity and reliability. Additionally, the study aimed at assessing its ability to predict the further need of instrumental assessment of swallowing by correlating its results with penetration aspiration scale and residue formation, in order to be used as a valid and reliable sensitive non-invasive screening tool deciding the need to further instrumental pediatric dysphagia assessment.

## Subjects and Methods

**Fig. 1:**
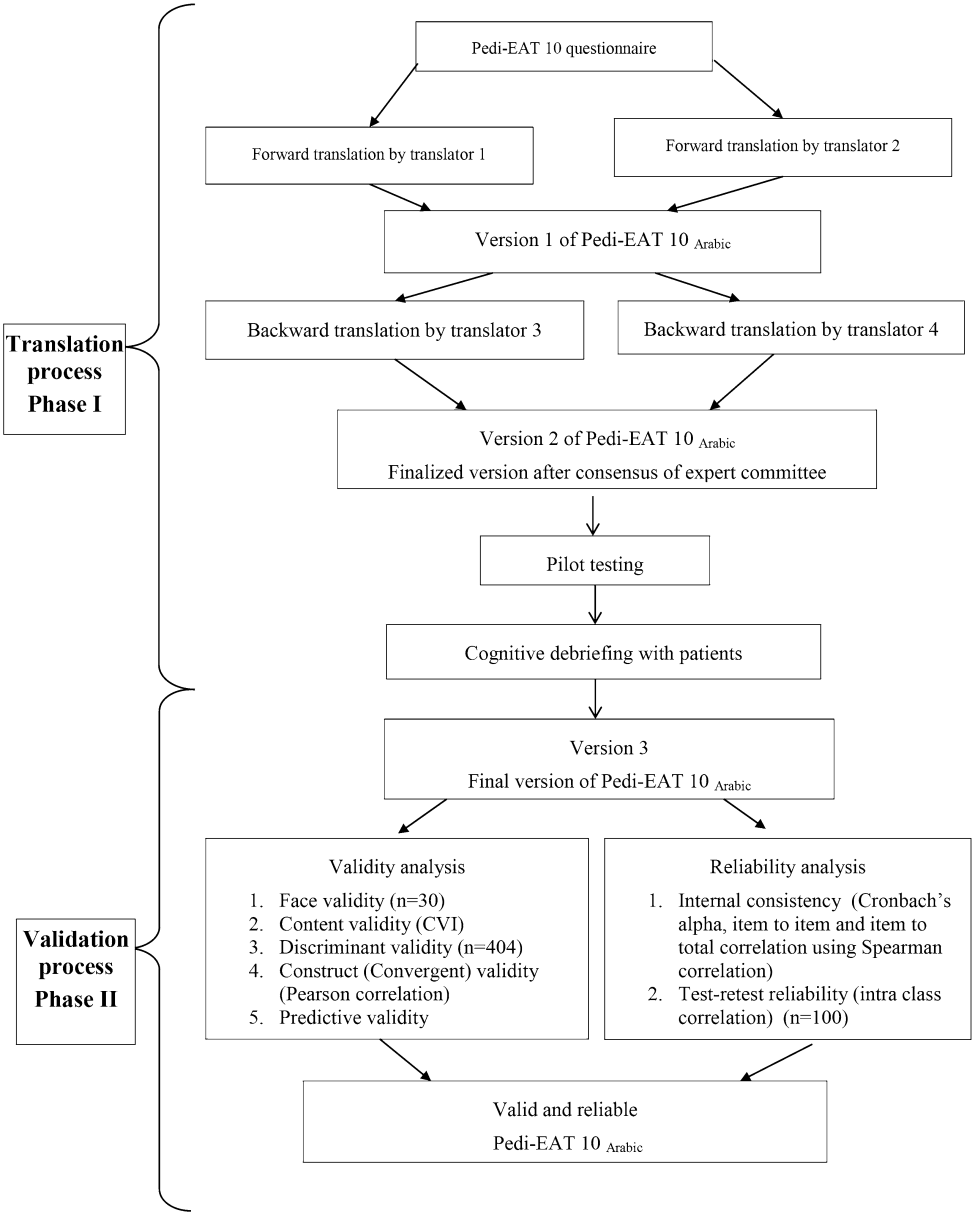
Research methodology flow chart

The research was conducted in three phases as shown in flow chart (Fig. 1).

### Phase 1: Arabic Translation of the English Pedi-EAT 10 Questionnaire Using Forward Backward Translation Method

Translation was carried out according to the principles of good practice for the translation and cultural adaptation process for patient reported outcome measures, as defined by the International Society for Pharmaco-economics and Outcome Research (ISPOR) Task Force for translation and cultural adaptation [21]. Initial forward translation from English to Arabic by two independent bilingual experienced phoniatricians, who are native Arabic speaking. A focus on conceptual equivalence instead of literal translation with avoiding slang or dialect terms or difficult unfamiliar terminology. Synthesis of a single ‘version 1’ by a third native speaker who revised the two forward translations, reconciled the differences and the three as a team decided on the ideal forward translation. The backward translation by two independent bilingual blinded to the original English version and having English as their first language; An expert committee review, composed of two expert phoniatricians (the original forward translators), a parent of a young child with swallowing problem and one of the expert translators who did the backward translation to evaluate the backward translation and consent on “version 2” of the translated questionnaire.

### Phase 2: Testing Face and Content Validity

#### Face Validity (Pretesting the Questionnaire)

The translated Arabic questionnaire was distributed to thirty caregivers of children attending the pediatric swallowing clinic, randomly selected, to ensure the representation of different levels of education. They were asked to fill the questionnaire. This step aimed at assessing comprehension and suitability of the questionnaire to different levels of education. Additional explanatory words were added to few questions because of some difficulties that were noticed in understanding the original items clearly. The final result was a culturally modified Pedi-EAT 10_Arabic_ “version 3”.

#### Content Validity

Three independent professors, experts in the area of research were asked to judge the relevance and clarity of each item of the questionnaire using 3-point relevance scale with a score ranged from 1 for highly relevant/ clear to 3 for not relevant/not clear.

### Phase 3: Validation and Reliability Testing of the Translated Questionnaire

A study was conducted including two groups:

- Two hundred and two (*n* = 202) children randomly selected from those attending swallowing clinic in phoniatrics unit, ORL department at Alexandria Main University Hospital from February 2019 to February 2020 complaining of dysphagia. Only caregivers who can read and write were included in the study (38 illiterate Arabic speaking caregivers were excluded). Mothers mainly answered the questionnaire with wide range of education from basic to high. The inclusion criteria of patients were pediatric age group above 6 months to be able to take both consistencies fluid and semisolid during the assessment, children who are orally fed and referred due to parents’ complaints about their child’s swallowing function but not admitted to a swallowing center before. The exclusion criteria were age below 6 months old, children who are non-fed orally, no parent complaints about their child’s swallowing function or children admitted to any swallowing center before for evaluation including our center. Minimum age in the studied group was 9 months old and maximum age was 10.08 years old.
- Another group of healthy children (*n* = 202) not complaining of dysphagia, no history of voice problem or laryngopharyngeal reflux disease, or any history of head and neck surgery or airway, neurologic, rheumatologic or neoplastic disorders, also no history of feeding difficulties, consistent coughing or chocking during meals, no genetic abnormalities or congenital heart disease were selected randomly from those attended the phoniatrics clinic due to other reasons such as delayed language development or siblings of patients attending the dysphagia clinic, after obtaining their caregiver’s consent. The control group was considered to be in the same age range of the studied patients to set a good reference to what is common in the swallowing of children not complaining of dysphagia. Minimum age was 9 months old and maximum age was 9.83 years old.
- Caregivers of both clinical cases and control children were administered the translated Arabic version of Pedi Eat 10 to fill it. To examine test–retest reliability of the translated questionnaire, 100 caregivers of patients with dysphagia were asked to refill the questionnaire after a two-week period following the first date without major therapeutic interventions taking place between the two times. Children who had to be on alternative mode of feeding due to aspiration diagnosed by instrumental assessment were not included in the test–retest sample to avoid bias of major therapeutic intervention. Fifty from the control group were invited to undertake the test 2 weeks later, while 65 of the dysphagia group were asked to refill the questionnaire as 15 of them had to start intervention immediately especially shifting to alternative mode of feeding.

For All Clinical Cases the Followings were Done

- Full history taking and oral motor examination followed by flexible endoscopic evaluation of swallowing (FEES) on the same day the caregivers filled the Pedi-EAT 10_Arabic_. Clinicians performing FEES were blinded to the results of Pedi-EAT 10_Arabic_ to avoid bias. A flexible digital video nasopharyngoscope (Storz video rhinolaryngoscope VP 11,101, Germany) was passed through the patient’s most patent naris.
- The standard FEES protocol was followed with slight modifications as swallowing was evaluated directly with six bolus challenges, three of each consistency (liquid and puree) of approximately 5 cc volume each, presented in the following order: Three boluses of pudding consistency (green-dyed yoghurt) followed by three thin liquid boluses (green-dyed 3% fat milk) given by bottle or cup according to developmental age. Solid food was not introduced but not included in our study as some children could not handle harder consistencies. Each bolus challenge was evaluated for the presence of penetration or aspiration, and was scored using the Penetration–Aspiration Scale (PAS)[22] and pharyngeal residue. Penetration was defined as PAS score from 2–5 and aspiration was defined as PAS score from 6–8. (The worst PAS out of all bolus challenges in all consistencies was used for analysis). Residue presence was scored first as a binary scale (yes or no) and also as a numeric score. A score of 0 was given if residue was absent in both consistencies tested and a score of 1 was given for each consistency in which any severity residue was observed with a maximal score of 2 if residue was present in both consistencies tested fluid and pudding.

### Statistical Analysis

#### Testing Questionnaire Reliability

The reliability of the Pedi Eat 10 _Arabic_ was assessed using internal consistency and test–retest reliability. Cronbach’s alpha, inter item and item to total correlation using spearman correlation were used for internal consistency. Values of Cronbach’s alpha ≥ 0.70 were considered satisfactory, and for item to total correlation, values > 0.20 were considered acceptable. The intra class correlation coefficient (ICC) was calculated to determine test–retest reliability. Values of ICC of > 0.80 indicate excellent agreement, good agreement with values from 0.61 to 0.80, moderate agreement with values from 0.41 to 0.60, and poor agreement with values < 0.40 [23, 24].

### Testing Questionnaire Validity

**Content validity:** The number of experts showed complete agreement for each item was divided by 3 (the total number of experts) to obtain item content validity index (I-CVI). Items CVI were added and divided by 10 to obtain total scale validity index (S-CVI). A minimum of 0.78 for I-CVIs and 0.8 for S-CVI/UA were recommended to consider the scale as having excellent content validity [25].

**Range of measurement** was based on the percentage of scores at the extremes of the scaling range, that is, the maximum possible score (ceiling effect) and the minimum possible score (floor effect). Tools with small floor or ceiling effects (1% to 15%) are considered to meet acceptable measurement standards.

**Discriminant validity** was determined utilizing the known-groups method. The known-groups method compares scale scores across groups known to differ in the health construct being investigated. It was assessed by comparing the total Pedi Eat 10 score among clinical cases and control group using Mann Whitney test. Significance was judged at 5% level of significance, comparing the response of groups to all items using Chi-square test.

**Construct (Convergent) validity:** It was assessed by Pearson correlation between the Pedi Eat 10 score and PAS score among children with dysphagia. Correlation coefficient gave an idea about the strength and direction of correlation.

**Predictive validity:** The ability of Pedi Eat 10 to predict penetration and residue formation was tested using Receiver Operating Characteristic (ROC) curve. Moreover, sensitivity and specificity with the most accurate cutoff values were calculated.

## Results

**Fig. 2:**
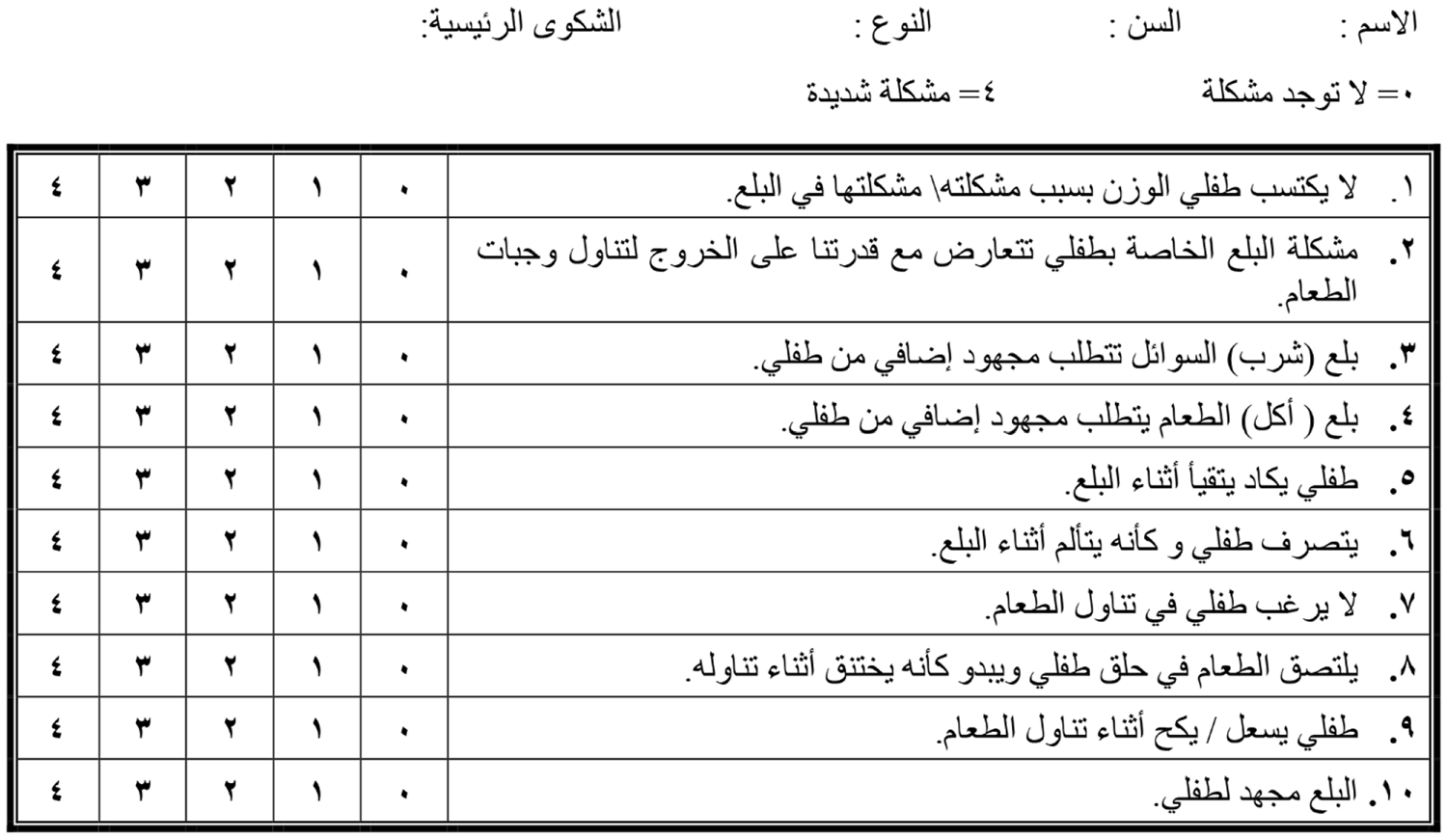
The Pedi-EAT10 _Arabic_ version

The translated Arabic version (Pedi-EAT10 _Arabic_) is presented in Fig. 2

### Description of Patients and Their Caregivers

Distribution of studied participants according to their age and sex showed no significant difference between healthy children and those with dysphagia as regards their age or sex as *P* > 0.05 as shown in Table 1. The mean age of dysphagia group was 2.48 ± 1.79 years versus mean 2.81 ± 1.79 years of the healthy group. 53.5% of the dysphagia group were males.

**Table 1:** Description of demographic characteristics of studied children

	Children with dysphagia (*n* = 202)	Healthy children (*n* = 202)	*P* value
Age in years			0.07
Mean ± SD	2.48 ± 1.79	2.81 ± 1.79	
min–max	0.75—10.08	0.75—9.83	
Gender			0.84
Males	108 (53.5%)	110 (54.5%)	
Females	94 (46.5%)	92 (45.5%)	
Mothers age			
Mean ± SD	33.0 ± 4.6	29.0 ± 5.2	0.01
min–max	19.0 – 43.0	21.0 – 37.0	

Mothers of the dysphagia group mean age at giving birth to this child was 33 ± 4.6 (min–max = 19–43 years old). While mothers of the healthy control group mean age at birth was 29 ± 5.2 with (min–max = 21–37).

**Fig. 3:**
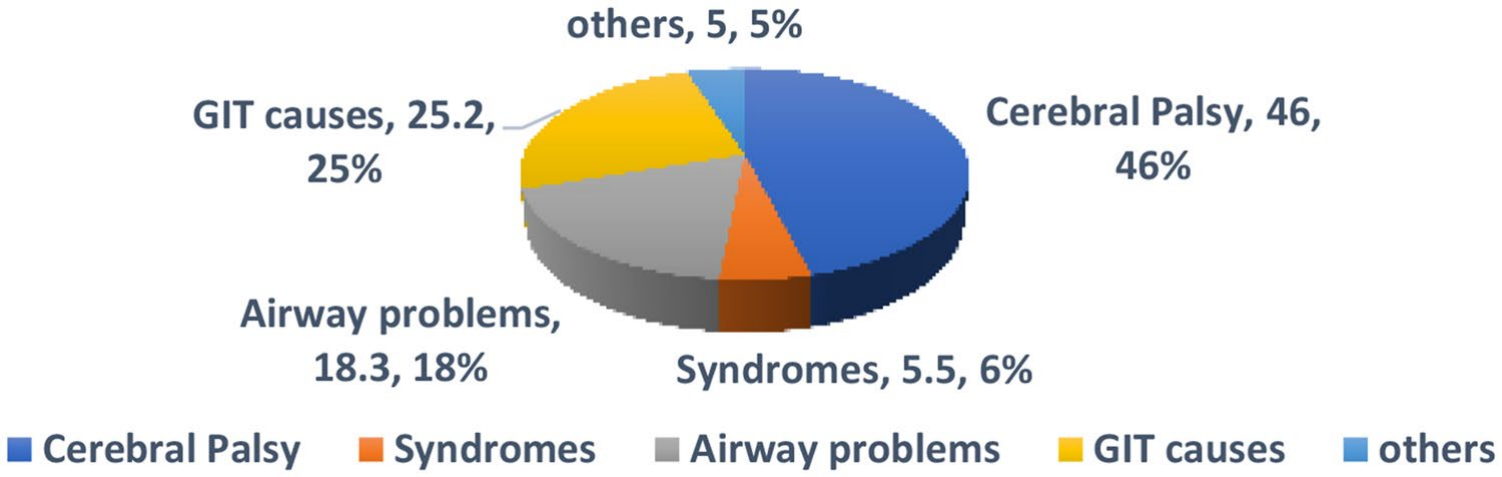
Etiological classification of dysphagia patients answering Pedi-EAT10 _Arabic_ questionnaire

Regarding the underlying disease among children with dysphagia, Fig. 2 shows that diagnoses including neurological disorders (cerebral palsy, vocal fold immobility), airway problems (laryngomalacia, laryngeal cleft, post TEF repair), gastrointestinal tract disorders, congenital heart disease and genetic disorders. (Fig. 3).

### Results of Phase II: Face and Content Validity of the Translated Arabic Version of the Questionnaire:

#### Face Validity:

The caregivers (*n* = 30) claimed that the statements were clear, easy to understand, and in a logical order. The only question that needed revision was question five due to lack of definitive culturally known translation for the word gag in Arabic, so further explanation of the word was written.

#### Content Validity:

Experts’ opinion regarding relevance of the questionnaire, the CVI for all items was 1 yielded a total CVI equal 1. Regarding clearance of the questionnaire, the CVI for all items was 1 except for item 5, it was 0.67 yielding a total CVI for clearance of 0.96. No missing cases in all items except for item 5 in which the percentage of missing cases was 15% and none from the control group. Regarding the floor effect among studied children, it ranged from 1.5 to 14.9%, however for the ceiling effect it was ranged from 24 to 53%.

### Results of Phase III: Reliability, Discriminant, Construct and Predictive Validity

#### Internal Consistency Reliability

Cronbach alpha, for the Pedi-EAT 10_Arabic_ questionnaire was 0.968 denoting excellent internal consistency reliability. The value of Cronbach’s alpha if an item deleted for the ten items ranged from 0.961 to 967 confirming excellent internal consistency of the questionnaire. Moreover the item to tem and item to total correlation using Spearman correlation denoting excellent reliability as shown in Table 2 (*r* > 0.2).

**Table 2:** Item to item and item to total correlation using Spearman correlation

	Item 2	Item 3	Item 4	Item 5	Item 6	Item 7	Item 8	Item 9	Item 10	Total
Item 1	.843	.719	.853	.769	.768	.707	.726	.776	.867	.912
Item 2		.718	.840	.759	.780	.697	.721	.776	.831	.906
Item 3			.682	.653	.628	.546	.699	.733	.758	.811
Item 4				.807	.779	.737	.734	.779	.871	.921
Item 5					.750	.712	.719	.713	.835	.878
Item 6						.667	.706	.755	.825	.869
Item 7							.650	.637	.684	.795
Item 8								.853	.783	.860
Item 9									.847	.892
Item 10										.945

All correlations were statistically significant at *P* < 0.01

#### Test–Retest Reliability

Table 3 shows adequate test–retest reliability for all questionnaire items with intra class correlation coefficient ranged from 0.97 to 1 **(excellent reliability).**

**Table 3:** Test–retest reliability using intra class correlation

Item	Item 1	Item 2	Item 3	Item 4	Item 5	Item 6	Item 7	Item 8	Item 9	Item 10	Total
Intra class correlation coefficient	1	1	0.998	0.998	0.995	1	0.972	0.973	0.998	0.988	0.998

#### Discriminant Validity

The total Pedi-EAT 10_Arabic_ score ranged from 13 to 40 among dysphagia cases with a median ± IQR = 27(21–34). On the other hand, the total score among healthy children ranged from 0 to 15 with a median ± IQR equals 0 (0–2). The difference between the two groups was statistically significant as *P* < 0.001. There was a significant difference between children with dysphagia and healthy children regarding their response to all items of the questionnaire with *P* < 0.001 using Chi-square test.

#### Construct (Convergent) and Predictive Validity

A significant strong positive correlation between Pedi-EAT 10_Arabic_ item score and **PAS score** was found with Pearson correlation coefficient *r* = 0.803 and *P* < 0.001.

**Fig. 4:**
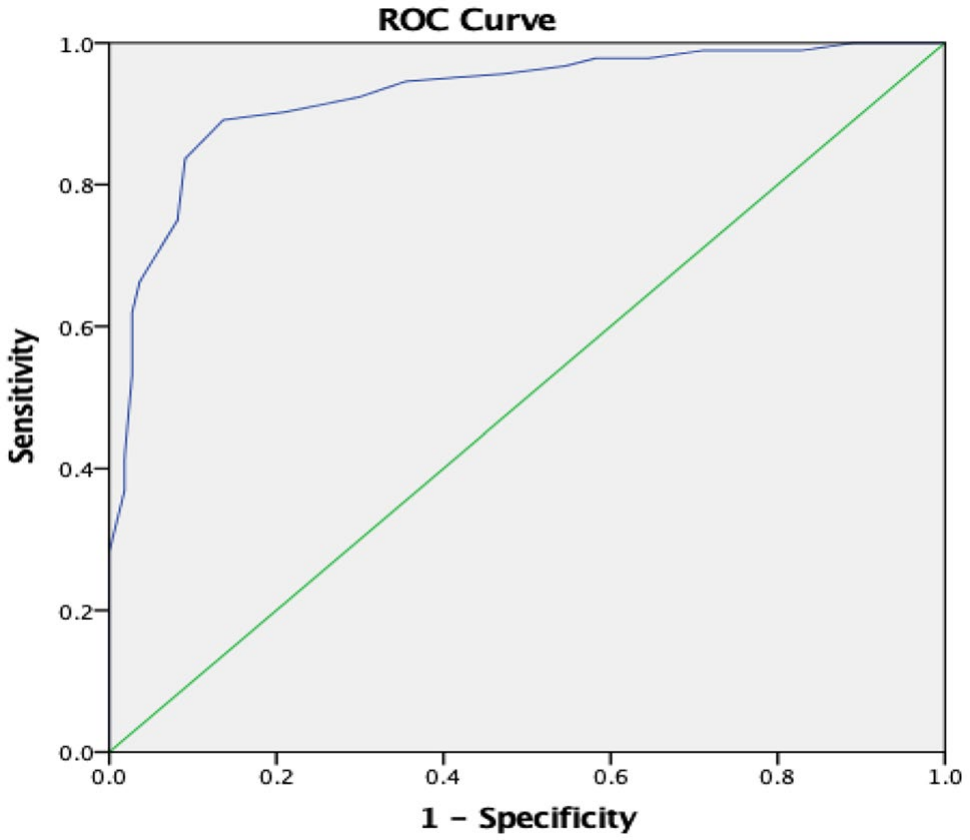
ROC curve showing accuracy of Pedi-EAT 10_Arabic_ questionnaire in predicting aspiration problems

Considering children with PAS score of 6, 7, 8 as having aspiration problem, out of 202 cases with dysphagia, 45.5% were having aspiration problem. Regarding, the mean Pedi-EAT 10_Arabic_ items score among those with aspiration problems was significantly higher than those with no aspiration (a mean of 33.3 ± 5.12 versus 22.0 ± 5.26) using student t test with *P* < 0.001.. ROC analysis for evaluating its diagnostic accuracy in predicting aspiration showed an AUC of 0.92 (95% CI of 0.89 to 0.96) and *P* < 0.001 (Fig. 4). At score higher than 28, the questionnaire has 89% sensitivity and 87.5% specificity, with positive and negative predictive values equal 84.5 and 90.5, respectively.

**Fig. 5:**
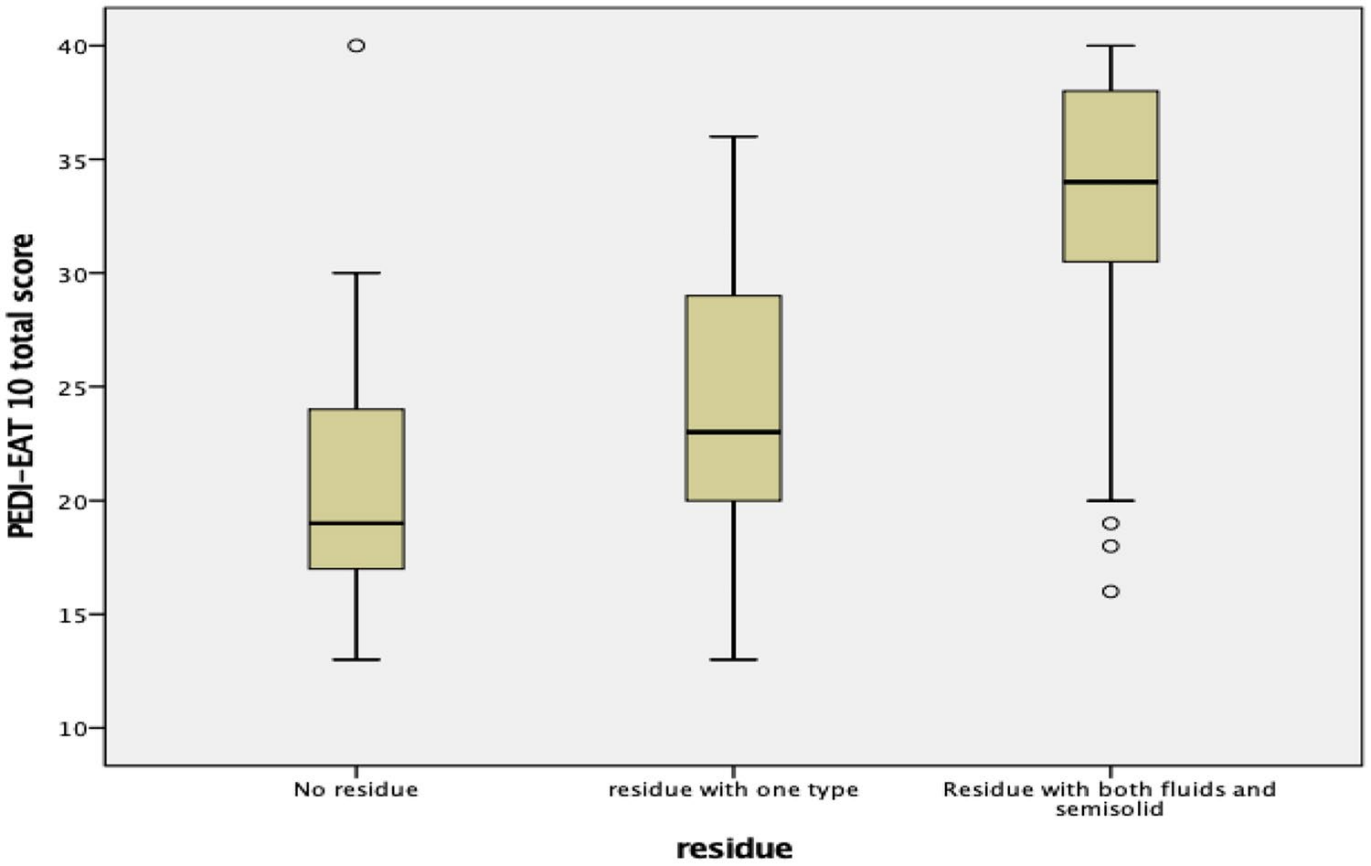
Box plot shows Pedi-EAT 10_Arabic_ scores in relation to residue score

**Regarding residue score**, there was a significant correlation between total residue score and Pedi-EAT 10_Arabic_ total score with *r* = 0.63 and *P* > 0.001. Nearly one half of children (48%) formed residue with either fluid or semisolid food (residue score of 1), 39.1% were having residue with both consistencies, however 12.9% formed no residue at all. A statistically significant difference between the three groups identified (no residue, residue with fluid or semisolid, residue with both) of children regarding the median Pedi-EAT 10_Arabic_ total score was found using Krauskal Wallis test with *P* < 0.001 (Fig. 5).

**Fig. 6:**
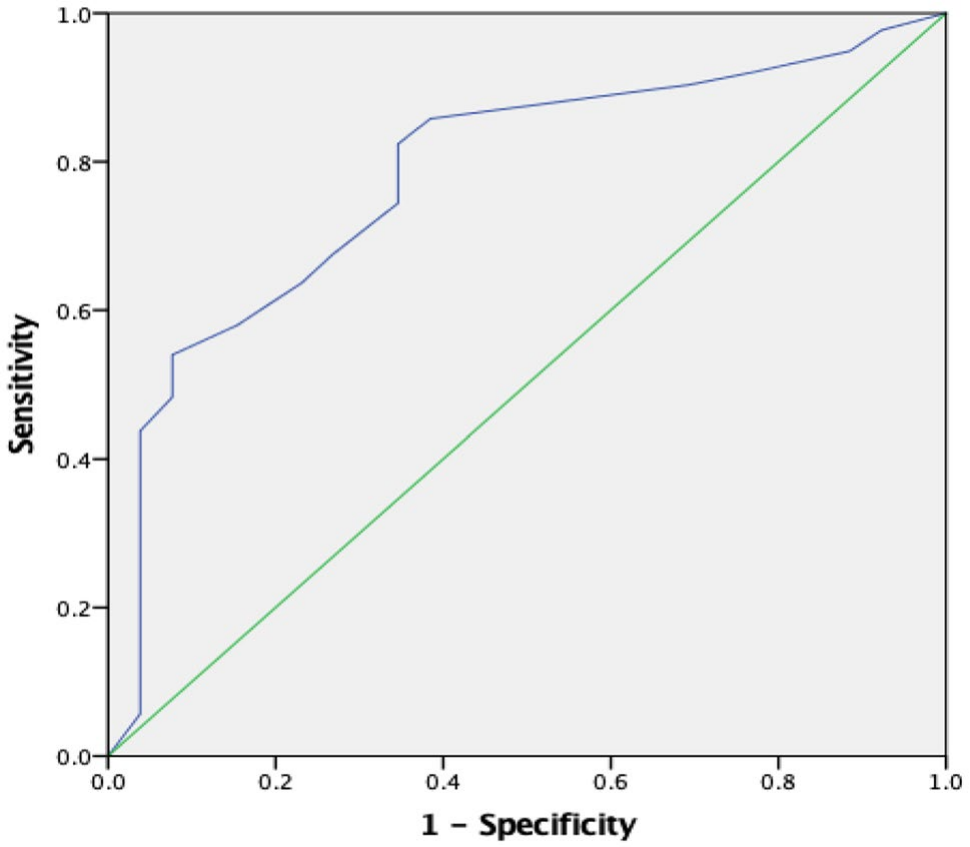
ROC curve showing accuracy of Pedi-EAT 10_Arabic_ questionnaire in predicting residue

Regarding accuracy of Pedi-EAT 10_Arabic_ score in predicting pharyngeal residue, the ROC analysis for evaluating accuracy of Pedi-EAT 10 in predicting residue formation showed an AUC of 0.78 (95% CI of 0.69 to 0.87) (Fig. 6).

## Discussion

Pedi-EAT 10_Arabic_ is a practical, simple to administer dysphagia caregiver symptom-specific outcome tool. It is a valid reliable non-instrumental assessment of pediatric patients at high risk of penetration-aspiration [15]. Considering the proved advantages of parent reported measures in pediatric population [26], Pedi-Eat 10 was originally adapted from EAT 10 [27],which has a translated valid Arabic version for adults [28]. Up to our knowledge, no current valid Arabic tool for pediatric age group, so this study aimed to provide an Arabic version of the Pedi-EAT10 questionnaire, validated to be used for research and clinic in Arabic speaking countries. This translated version, after transcultural adaptation and validation has proven to be a discriminant, valid and reliable tool to screen for dysphagia and assess the need of further instrumental assessment of swallowing. To provide equivalence between the Arabic and the English version of the Pedi-EAT 10, a rigorous translation and Cross-cultural adaptation processes was followed. Proof of Correctness and equivalence between the two questionnaires was provided by the high internal consistency of the translated questionnaire, by its consistent construct validity and the excellent test–retest reliability observed in results.

The mean age of the studied population was 2.48 ± 1.79 years, min–max (0.75—10.08) while the mean age for healthy children in this study was 2.81 ± 1.79 years, min–max (0.75—9.83) of which 54.5% were males. The mean age of the healthy children in the original PEDI-EAT 10 was 4.4 (± 1.5) years (min ¼2, max ¼8), of which 51% were male [15]. So our study had wider age range recruited both younger and elder than original study with lower mean age.

The pediatric dysphagia patients showed different etiologies predominantly neurogenic followed by GIT patients, at the expense of airway structural problems which can affect generalization to other patients. However, the original Pedi-EAT 10 was validated by comparing healthy to children with cerebral palsy (CP) [15]. Another study done in 2017 showed that Pedi-EAT 10 as a valid tool in predicting aspiration in children with esophageal atresia [29].

The total content validity of the translated Arabic version was of 0.96 so it was very close to the CVI of original version which was 0.91 [15].

Pedi-EAT 10_Arabic_ was proved to be reliable by high internal consistency (Cronbach's alpha of 0.986), mildly higher than original Cronbach alpha for test retest was 0.87 [15]. Item to item and item to total correlation coefficient ranged from 0.546 to 0.94 denoting significant moderate to strong correlation. Adequate test–retest reliability for all questionnaire items with intra class correlation coefficient ranged from 0.97 to 1 (excellent) and total ICC = 0.998, which is similar to the results of the original English version, with total ICC 0.983 at 95% confidence interval [15].

In this cross-sectional observational study, **discriminant validity** of the Pedi-EAT 10_Arabic_ scores was established on a group of 202 pediatric patients with dysphagia and 202 healthy, median of dysphagia group was median ± IQR = 27(21–34) versus 0 (0–2) for the healthy group with statistically significant difference between both groups. In the original version. The mean PEDI-EAT-10 score of the normal cohort was significantly lower than the mean PEDI-EAT-10 scores of the CP children who had complaints about swallowing difficulty (z ¼ 10.01, *P* < 0.001) [15].

The **construct (convergent) validity** was proofed by symptom-specific questionnaire Pedi-EAT 10_Arabic_ and the characteristics of pharyngeal dysphagia identified using FEES in dysphagic patients was described mainly in terms of PAS score and pharyngeal residue [29, 30]. A strong positive correlation between Pedi-EAT 10_Arabic_ item score and PAS score with Spearman correlation coefficient *r* = 0.803 and *P* < 0.001. Spearman correlation showed significant correlation between residue score and Pedi-EAT 10_Arabic_ total score with *r* = 0.63 and *P* > 0.001. Mean Pedi-EAT 10_Arabic_ scores were significantly higher among those aspirating than patients not aspirating. These similar results were reported by the original Pedi-EAT 10_Arabic_ tool which was proven to be a consistent scale for determining the risk of penetration/ aspiration in children with CP tested with VFSS and is used as a discriminative tool for identification of penetration/ aspiration risk in children and higher scores indicate a greater risk for penetration/ aspiration [15].

Regarding the **predictive validity** of the Pedi-EAT 10_Arabic_, the cut off point for detection of aspiration with higher sensitivity than specificity was more than 28 with 89% sensitivity and 87.5% specificity with positive predictive value (PPV) 84.5% and negative predictive value (NPV) 90.5%. In a study done in 2018, the sensitivity of Pedi-EAT-10 score greater than 12 in predicting aspiration in children with neurogenic impairment was 77% and the specificity was 54%, has a PPV of 69% and a NPV of 64% [31]. While in another study sensitivity and specificity of Pedi-EAT 10 to predict aspiration in children with esophageal atresia with cut off score 7 was 88% and 77% and the PPV and NPV were 22% and 11%, respectively [29]. Possible explanation for the gap in cut off value for aspiration and the discriminatory capacity can be cultural variation as the mean of Pedi-EAT 10_Arabic_ was 3.82 ± 3.53 for healthy children and 26.70 ± 7.70 for children with dysphagia, the mean score of Pedi-EAT 10 tool for healthy children and children with cerebral palsy was 0.26 ± 1.83 and 19.5 ± 11, respectively, so even healthy controls had higher scores. Another explanation is the different etiologies: esophageal versus oropharyngeal esophageal dysphagia patients will have Pedi-EAT 10_Arabic_ scores similar to those with oropharyngeal dysphagia, but lower PAS scores than oropharyngeal dysphagia patients. CP children mostly with oropharyngeal dysphagia were around half of the patients and most of the other patients showed mainly esophageal dysphagia, but pharyngeal dysphagia was also not absent in these patients. The results of original Pedi-EAT 10 score of 4 or higher was classified abnormal with high sensitivity and specificity in predicting penetration/ aspiration on instrumental swallowing study [15].

Since post swallow pharyngeal pooling may be a risk factor for tracheal aspiration [30] was estimated by FEES. A significant correlation was found in our study between residue score and Arabic Pedi-EAT 10 total score. These results were similar to those reported by residue assessment by FEES but in the adult form of the questionnaire EAT 10 [32].

Our study has several limitations: exclusion of illiterate patients was made to decrease bias of the language complexity, especially the fact that it is written in formal Arabic language not in Egyptian dialect.

While ensuring the patients included in the study did not undergo major therapeutic interventions during the 2-week test retest interval, some had to change their mode of feeding from oral to NGT when aspiration was proved by instrumental assessment so these were excluded from the test retest assessment.

Lastly Pedi-EAT 10_Arabic_ was not tested in our study for responsiveness, i.e., the ability of the caregiver reported outcome measurement to reflect change after intervention, though this psychometric property has been demonstrated in other studies [27, 31, 33]. The ability of questionnaire to reflect after intervention which can be tested in future studies.

## Conclusion

Arabic Pedi-EAT 10_Arabic_ is a reliable and valid care giver reported outcome measure for Arabic speaking caregivers of pediatric dysphagia patients. It has a strong correlation with objective findings on ISA, including penetration, aspiration and residue. As a potential screening tool for the need of further instrumental assessment of swallowing. Its discriminatory capacity of pharyngeal dysphagia was found to be high as described in terms of aspiration and post swallow residue.

## Abbreviations

Pedi-EAT 10: Pediatric Eating Assessment tool 10 questionnaire
Pedi–EAT 10_Arabic_: Arabic version of pediatric Eating Assessment tool questionnaire
ORL: Oto Rhino Laryngology
ISA: Instrumental Swallowing Assessment
FEES: Flexible endoscopic evaluation of swallowing
VFSS: Video fluoroscopic swallowing study
ASHA: American speech and hearing association
CMS: Centers of Medicare and Medicaid services
ISPOR: International society of pharmaco economics and Outcome research
PAS: Penetration Aspiration Scale score
RES: Residue
CVI: Content validity index
I-CVI: Item Content Validity Index
S-CVI/UA: Scale-Level Content Validity Index Universal Agreement
AUC: Area under the curve
IQR: Interquartile range
PPV: Positive predictive value
NPV: Negative predictive value
CP: Cerebral palsy
PCR: Polymerase chain reaction test
Min: Minimum
Max: Maximum
ROC: Receiver Operating Characteristic curve
